# On the use of beam precession for serial electron crystallography

**DOI:** 10.1107/S1600576725005606

**Published:** 2025-07-25

**Authors:** Sergi Plana-Ruiz, Penghan Lu, Govind Ummethala, Rafal E. Dunin-Borkowski

**Affiliations:** a Servei de Recursos Científics i Tècnics Universitat Rovira i Virgili Avinguda Països Catalans 26 Tarragona Catalonia43007 Spain; b LENS-MIND, Department of Electronics and Biomedical Engineering Universitat de Barcelona Martí i Franquès 1 Barcelona Catalonia08028 Spain; c Ernst Ruska-Centre for Microscopy and Spectroscopy with Electrons Forschungszentrum Jülich Wilhelm-Johnen-Strasse Jülich52425 Germany; DESY, Hamburg, Germany

**Keywords:** precession electron diffraction, serial crystallography, SerialED, 3D ED, scanning transmission electron microscopy

## Abstract

During the past few years, serial electron crystallography (serial electron diffraction) has been gaining attention for the structure determination of crystalline compounds that are sensitive to irradiation by an electron beam. By recording a single electron diffraction pattern per crystal, indexing thousands to tens of thousands of such patterns and merging the reflection intensities of the successfully indexed patterns, one can retrieve crystal structure models with strongly mitigated beam damage contributions. However, one of the technique’s bottlenecks is the need to obtain so many well indexed diffraction patterns, which leads to the collection of raw diffraction data in an automated way that usually yields low indexing rates. This work demonstrates how to overcome this limitation by performing the serial crystallography experiment following a semi-automated routine with a precessed electron beam (serial precession electron diffraction). The precession movement increases the number of reflections present in the diffraction patterns, and dynamical effects related to specific orientations of the crystals with respect to the electron beam are greatly minimized. This leads to more uniform reflection intensities across the serial data set, and a smaller number of patterns are required to merge the reflection intensities for good statistics. Furthermore, structure refinements based on the dynamical diffraction theory become possible due to the diffraction volume integration of beam precession, providing a novel approach for more accurate structure models. In this context, the use of beam precession is presented as an advantageous tool for serial electron crystallography, as it enables reliable crystal structure analysis with a lower amount of diffraction data.

## Abstract 2

The use of electron beam precession in a (scanning) transmission electron microscope for serial crystallography experiments is thoroughly investigated, showing its potential for better crystal structure determination and refinement in the context of 3D electron diffraction.

## 1. Introduction

The field of serial crystallography aims at studying crystal structures via a collection of diffraction patterns, each of which corresponds to a randomly oriented single individual crystal. This methodology was primarily developed at X-ray free-electron lasers (XFELs) as a novel tool to study sub­micro­metre-sized macromolecular crystals at the highest resolutions in space and time, overcoming one of the hindrances of biomolecular imaging at earlier times (Neutze *et al.*, 2000 ▸; Chapman *et al.*, 2011 ▸; Pellegrini, 2012 ▸). The use of a very bright X-ray beam pulsed at the femtosecond scale enables the illumination (and disintegration) of crystals that are injected into the X-ray optical path in a constant stream, capturing the diffraction signal produced by each hit (Spence, 2017 ▸). The analysis of the resulting thousands to hundreds of thousands of effective diffraction patterns allows the elucidation of structures from crystals too small to be revealed by more conventional methods (Colletier *et al.*, 2016 ▸), as well as the dynamics of protein nanocrystals (Nass *et al.*, 2020 ▸). However, the XFELs or synchrotron beamlines prepared for such experiments (Mehrabi *et al.*, 2021 ▸) are not readily available to most laboratories, high crystal densities are required per sample, and there is significant sample waste from the jet delivery system. In this context, a serial crystallography experiment in a transmission electron microscope appears as an alternative and likely solution because electrons can be used to obtain diffraction data from individual and user-selected nanocrystals, and their interaction with matter is stronger than that of X-rays or neutrons, which leads to a higher probability of elastic scattering events and a smaller energy deposition by inelastic interactions (Henderson, 1995 ▸; Clabbers & Abrahams, 2018 ▸).

Serial electron crystallography deals with electron diffraction (ED) patterns that do not have an *a priori* orientational relation between them. The first realization was done by using the transmission electron microscopy (TEM) operation mode of the microscope (Smeets *et al.*, 2018 ▸), and later it was extended to the scanning transmission electron microscopy (STEM) mode (Bücker *et al.*, 2020 ▸). Essentially, the data acquisition workflow is the same iterative routine: an acquisition of a (S)TEM reference image for possible crystalline targets, automated or manual selection of electron beam positions, collection of an ED pattern for each chosen point through a single exposure or a serialized frame acquisition, and shifting of the stage to another interesting area. By following this protocol, the whole electro-transparent area of a typical TEM grid can be inspected and thousands of patterns can be collected within a day. Afterwards, the data processing takes place through custom or adapted versions of pipelines from XFELs (Bücker *et al.*, 2021 ▸) that include finding of the central beam and reflection positions (peaks) for each pattern, indexing with usually known unit-cell parameters [some options exist for unknown cells (Belletti *et al.*, 2000 ▸; Jiang *et al.*, 2011 ▸; Gevorkov *et al.*, 2019 ▸; Gorelik *et al.*, 2025 ▸)], reflection intensity extraction from the successfully indexed patterns, and merging of the extracted intensities.

One of the requisites for serial crystallography is a high number of diffraction patterns. The same requirements apply to SerialED, where several hundreds to tens of thousands of patterns have been reported (Smeets *et al.*, 2018 ▸; Bücker *et al.*, 2020 ▸; Nikbin *et al.*, 2024 ▸). However, during the 1990s, several studies showed that the use of a few zone-axis ED patterns (ED patterns oriented along high-symmetry axes) was enough to determine crystal structures (Morniroli & Steeds, 1992 ▸; Nicolopoulos *et al.*, 1995 ▸; Dorset, 1996 ▸, 1997 ▸). One of the disadvantages of this methodology was the time-consuming orientation of the crystals and the consequent unavoidable illumination of crystals before any meaningful acquisition, which is critical for beam-sensitive specimens. Furthermore, avoiding the effects of dynamical diffraction required very thin samples, and even then these were not fully diminished as they are enhanced when the crystal is oriented along the zone axis. In this context, the combination of zone-axis ED patterns and high-resolution TEM images helped to push the accuracy of structure models characterized by electrons (Weirich *et al.*, 1996 ▸, 2006 ▸), but one of the biggest steps was the acquisition of diffraction data by beam precession, also known as rocking illumination (Vincent & Midgley, 1994 ▸).

Precession electron diffraction (PED) was invented to average out the non-systematic dynamical effects, such as Kikuchi lines, double diffraction or diffuse scattering, which are highly dependent on the crystal orientation, and render diffraction patterns with pseudo-kinematical reflection intensities, *i.e.* reflection intensities that resemble more closely the respective ones calculated by the kinematical theory of diffraction. Crystal structure analysis from zone-axis PED patterns made crystal structure analyses easier (Weirich *et al.*, 2006 ▸; Gemmi & Nicolopoulos, 2007 ▸; Sinkler *et al.*, 2007 ▸). Later on, the idea of three-dimensional electron diffraction (3D ED) was introduced (Kolb *et al.*, 2007 ▸, 2008 ▸; Gemmi *et al.*, 2019 ▸): the collection of non-oriented ED patterns from a single nanocrystal at subsequent and usually equidistant tilts of the sample holder. Here, the addition of precession also resulted in a significant improvement (Mugnaioli *et al.*, 2009 ▸) as the ranking of reflection intensities was better preserved for *ab initio* structure solutions (Klein & David, 2011 ▸; Eggeman & Midgley, 2012 ▸) and subsequently enabled dynamical refinements (Palatinus *et al.*, 2013 ▸). From another perspective, the addition of precession to scanning electron diffraction (SED), known as 4D-STEM in other literature (Ophus, 2019 ▸), also resulted in better results, for instance for phase and orientation mapping (Viladot *et al.*, 2013 ▸). The enhanced quality of these maps comes from the wobbling of the Ewald sphere by beam precession since it integrates a larger volume in the diffraction space, which leads to more reflections per ED pattern with fewer dynamically related contributions. In this way, indexing algorithms like template matching work better (Rauch & Dupuy, 2005 ▸; Rauch *et al.*, 2010 ▸). Other SED applications like strain mapping (Cooper *et al.*, 2015 ▸) or electric field mapping (Lorenzen *et al.*, 2024 ▸) also benefit from precession, but in this case the advantage is related to the uniformization of the intensity inside the reflection discs.

Given the history of success for PED, the present work aims to evaluate the benefits of precession in the context of serial electron crystallography for crystal structure determination and refinement. The analysis described here is performed from ED patterns acquired with and without precession from a beam-stable sample under different microscope setups and processed with different software. In this way, a detailed and fair comparison of the retrieved and refined structure models between static and precessed serial data is presented.

## 2. Materials and methods

BaSO_4_ (baryte) was used for the comparisons between statically acquired (static) and precession-integrated (precessed) serial data, referenced in this work as SerialED and SerialPED, respectively. Baryte is an inorganic material crystallizing in an orthorhombic space group (8.879 Å, 5.454 Å, 7.154 Å; *Pnma*) up to very high resolutions (more than 2 Å^−1^ / 0.5 Å) with five atoms in the asymmetric unit (Jacobsen *et al.*, 1998 ▸). Barium, sulfur and two of the oxygen atoms (O1 and O3) lie on the mirror plane (4*c*), while the third oxygen atom (O2) is located on a general site (8*d*). Electron irradiation does not diminish its crystalline state, hence its use as a reference material in other ED reports (Mugnaioli *et al.*, 2009 ▸; Plana-Ruiz *et al.*, 2020 ▸). A fine powder of this compound was purchased from Merck, dispersed in ethanol and cast onto typical Cu TEM grids (ultra-thin continuous amorphous C).

The acquisition of the static and precessed SerialED data was performed with two different transmission electron microscopes equipped with two different detectors at room temperature. The first was a JEOL F200 with a cold field-emission electron gun operated at 200 kV (0.02508 Å) in STEM mode (probe size 8, 10 µm condenser aperture) and a post-column Gatan OneView camera (16-bit CMOS-based and optical fibre-coupled detector of 4096 × 4096 pixels, 15 µm physical pixel size). The STEM operation mode was chosen instead of the TEM mode because it is better suited to diffraction experiments, as already reported (Kolb *et al.*, 2019 ▸; Hogan-Lamarre *et al.*, 2024 ▸). A quasi-parallel beam in STEM was manually aligned following the routine established by Plana-Ruiz *et al.* (2018 ▸), so that the most parallel condition (0.09 ± 0.02 mrad of convergence semi-angle at FWHM) could be attained with a beam diameter of 200 nm (FWHM) with an electron flux density of 30 e^−^ Å^−2^ s^−1^ for the diffraction pattern acquisition (exposure time of 0.25 s, electron fluence of 8 e^−^ Å^−2^). Small convergence semi-angles are desired for ED patterns in order to obtain statistically significant counts for the weakest reflections. A more con­vergent electron probe was used for the STEM reference images acquired with a JEOL high-angle annular dark-field (HAADF) detector. A Gatan *Digital Micrograph* script was developed to facilitate the data collections with a graphical user interface (GUI) for ease of use (Fig. S1 in the supporting information). Briefly, it allows one to save and retrieve beam conditions for imaging and diffraction settings (beam size, camera length and projector coil offsets), acquire STEM reference images, and manually select as many beam positions as one wishes from this reference, and it automatically shifts the beam and collects and stores the ED patterns (freely available at https://github.com/sergiPlana/TEMEDtools/tree/main/STEMSerialED). The precession of the electron beam at 100 Hz frequency was enabled by a P2000 prototype precession unit provided by NanoMEGAS SPRL. Precession-assisted 3D ED tilt-series data were automatically collected using the Fast-ADT module with a JEOL tomography holder that allows a maximum tilt range of ±70° (Plana-Ruiz *et al.*, 2020 ▸). The second microscope used in this work was a TESCAN Tensor, a STEM-dedicated Schottky FEG operated at 100 kV (0.03701 Å) with a Dectris Quadro detector (16-bit hybrid-pixel direct electron detector of 512 × 512 pixels, 75 µm physical pixel size). The interface *ExpertPI* (TESCAN)based on Python v3.11.6 was used for rapid switching between the two different beam settings, the acquisition of STEM bright-field (BF) reference images, and the acquisition of precessed and static diffraction patterns from manually selected positions. A 200 nm beam diameter with 0.38 ± 0.04 mrad of convergence semi-angle (FWHM) and an electron flux density of 20 e^−^ Å^−2^ s^−1^ was set for the collection of the patterns (exposure time of 100 ms, electron fluence of 2 e^−^ Å^−2^). Precession of the electron beam was enabled at a frequency of 72 kHz from the signal unit integrated into the microscope.

**Figure 1:**
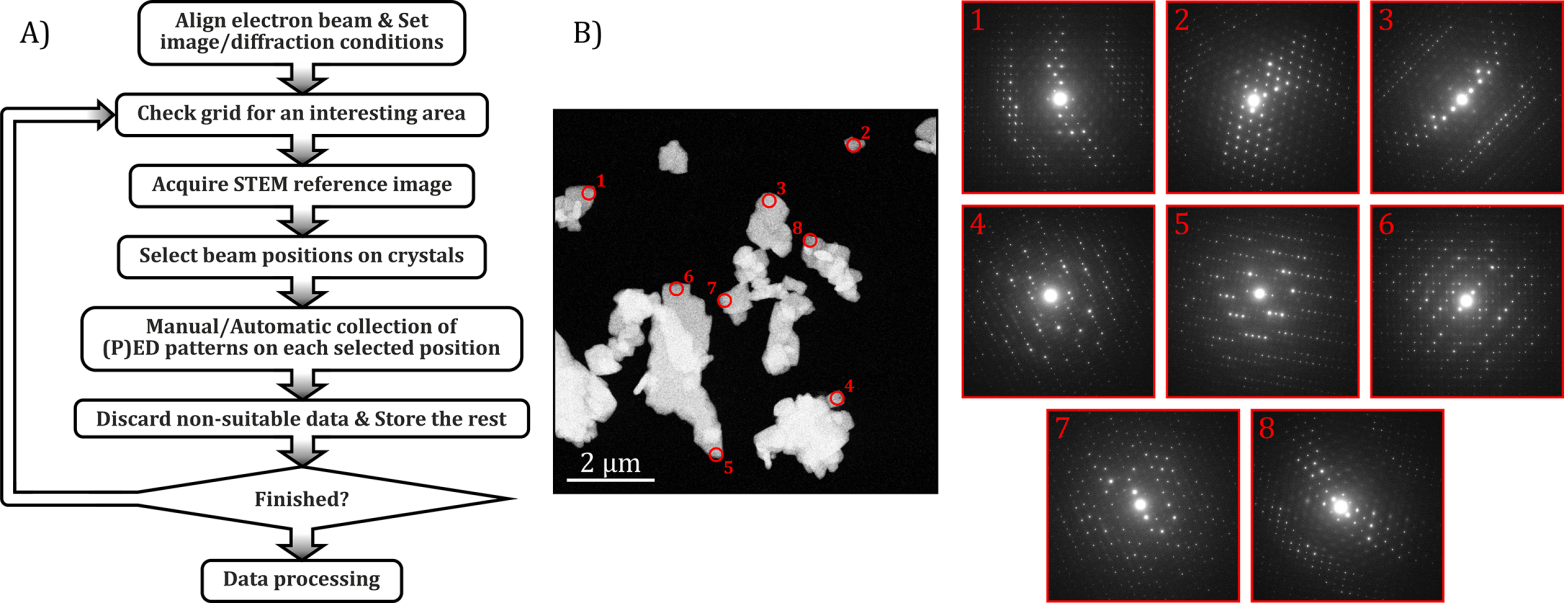
(*a*) Schematic diagram of the semi-automated Serial(P)ED experiment workflow. (*b*) An example of a STEM-HAADF reference image and the respective PED patterns acquired at each of the areas marked in red.

Fig. 1 ▸ shows the acquisition workflow followed in this study. First, the two beam settings are stored from the different microscope software programs so that they can be recalled automatically between imaging and diffraction. The *z* height is adjusted to have a focused scanned image of an electro-transparent area and precession is aligned in this condition. Then, an area with plenty of crystals is sought with the imaging setting. Once a suitable region has been found, precession is activated, and the *z* height is adjusted if needed in order to minimize the fringing generated by the precession movement on the scanned image. This ensures that the pivot point of the precession movement is precisely on the specimen plane and the region of interest is in focus at the same time. Subsequently, a reference STEM image is collected and kept on the PC screen to select the areas from which ED patterns will be acquired. The criterion used to select a suitable position was a thin area of non-overlapping individual crystals. In the JEOL F200, several positions were manually selected at once for each reference image, and the diffraction patterns were automatically collected from these and displayed in the workspace of *Digital Micrograph*. The ones that looked too thick because of a high inelastic background contribution were discarded by closing them and the rest were automatically stored in a preset folder. In the TESCAN Tensor, each diffraction pattern was collected manually by placing the beam on an interesting area on an individual basis. For both microscopes, each pattern acquisition was repeated twice, with and without precession. Once an area had been completely sampled, another region was sought to repeat this whole procedure until enough patterns were obtained.

Different software packages were employed to process the diffraction data. Data reduction from raw frames to reflection intensity (.hkl) files was independently obtained from two pipelines for comparison purposes: *PETS2* (Version 2.2.20240601; Palatinus *et al.*, 2019 ▸) and the *diffractem* Python package (Version 0.4.0; Bücker *et al.*, 2021 ▸) which uses routines from the *CrystFEL* software suite (Version 0.10.0; White *et al.*, 2012 ▸). For the latter, the indexing was retrieved by the *pinkIndexer* algorithm (Gevorkov *et al.*, 2020 ▸) available from the *indexamajig* program of *CrystFEL*, and the merging of all integrated reflection intensities was done via the *partialator* program of *CrystFEL*, which includes scaling, different models for the calculation of partial intensities and post-refinement (White, 2014 ▸). *Ab initio* structure solutions were obtained by direct methods in *SIR2014* (Version 17.10; Burla *et al.*, 2015 ▸) using the *BEA* algorithm to improve the resulting structure models (Luca Cascarano *et al.*, 2010 ▸) and the charge-flipping algorithm in *SUPERFLIP* (Version 09.21.20; Palatinus & Chapuis, 2007 ▸). Crystal structure refinements were done with *JANA2020* (Version 1.3.57; Petříček *et al.*, 2023 ▸). Dynamical refinements were executed in *JANA2020* using the *dyngo* module (Palatinus, Petříček & Corrêa, 2015 ▸). *XPREP* (Version 2014/02; Karplus & Diederichs, 2015 ▸) was used for the calculation of *R*_rim_ to have a symmetry fulfilment indicator independent of reflection multiplicity. Dynamical reflection intensities were calculated from *dyngo* using wrap-up Python scripts (Cabaj *et al.*, 2024 ▸). Visualizations of the structure models were obtained from *VESTA3* (Momma & Izumi, 2011 ▸).

## 3. Results

**Figure 2:**
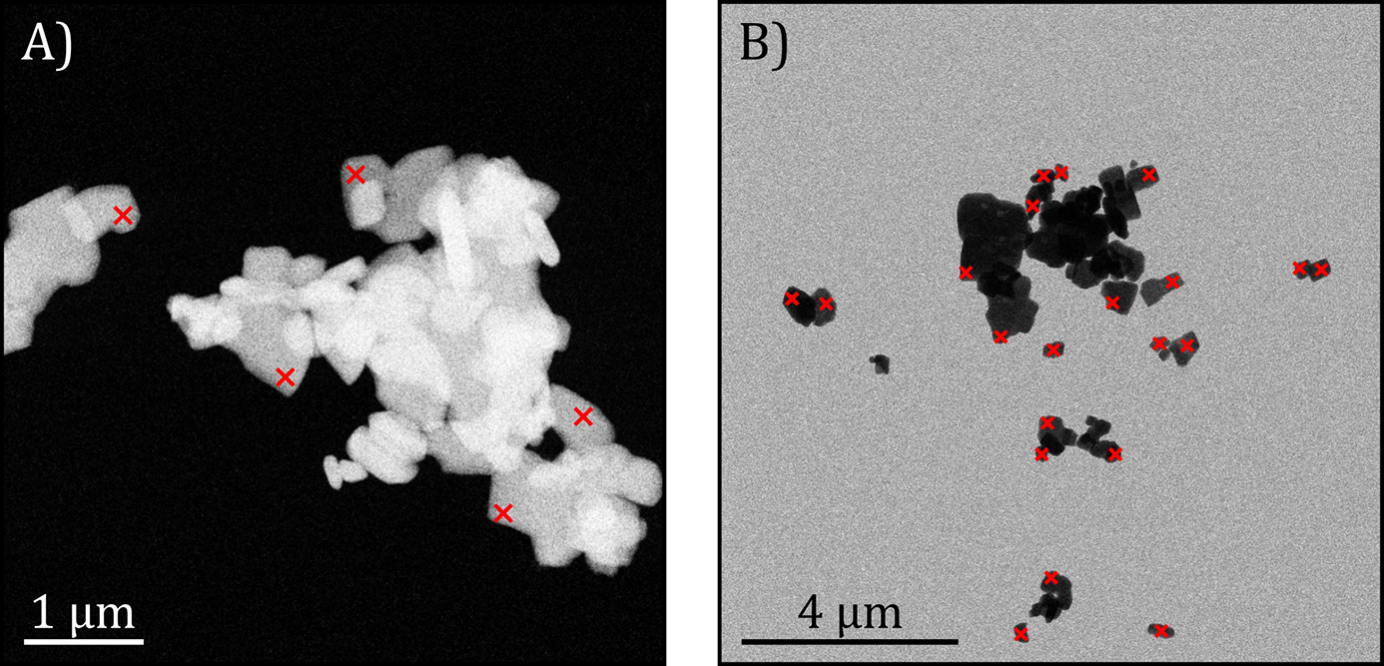
Representative reference images used to select interesting BaSO_4_ crystallites. The red crosses correspond to the positions where the beam was placed to acquire ED patterns. (*a*) STEM-HAADF image from the JEOL F200 TEM at 200 kV. (*b*) STEM-BF image from the TESCAN Tensor microscope at 100 kV.

Two hundred baryte crystals were measured with and without 0.92° of precession integration across 45 reference images with fields of view between 2.7 × 2.7 µm and 8.7 × 8.7 µm with the F200 TEM in ∼1.5 h. Additionally, 495 crystals were inspected with and without 0.97° of precession from 38 reference areas of 12.5 × 12.5 µm with the Tensor microscope in ∼3 h. The precession angles were chosen to exceed the Bragg angle of the maximum resolution or the highest-order reflection used for crystal structure analysis (here 0.5 Å), as well as to avoid reflection overlapping from higher-order Laue zones and optical distortions of highly tilted beams (Midgley & Eggeman, 2015 ▸). Since the two sets of serial diffraction data come from different electron energies and electron optics, and the reflection intensities were also detected from two different technologies, their processing was done separately. Fig. 2 ▸ shows representative reference images from both setups for crystal measurement selection.

Two software packages were used for data reduction to compare two different ways of extracting reflection intensity and two different indexing procedures. The *PETS2* package offers the possibility of extracting reflection intensities based on the fitting of specific functions to the shape of the rocking curves. For indexing, a template-matching algorithm has been added recently that assigns orientation angles with respect to a reference orientation matrix for each pattern as if it were a 3D ED data set and enables it to be processed likewise (Palatinus *et al.*, 2023 ▸). On the other hand, *diffractem*/*CrystFEL* offers the possibility of considering the partiality of reflection intensities from different geometric models for intensity extraction, *i.e.* that the integrated intensity from a given reflection does not come from the Bragg condition (White, 2014 ▸). From the indexing perspective, the algorithm of *pinkIndexer* is presented as an alternative to parameterize the possible orientations of the crystal lattice as curves in a 3D rotation space, which has been successfully tested on X-ray and electron diffraction (Gevorkov *et al.*, 2020 ▸; Bücker *et al.*, 2020 ▸; Hogan-Lamarre *et al.*, 2024 ▸). Table 1 ▸ shows some of the statistics of the resulting data reduction process from the Serial(P)ED experiments using these two processing pipelines.

An important point for data reduction is to determine if the found crystal orientations (indexing) are correct. When dealing with thousands or even millions of diffraction patterns, filters are available to discard the incorrectly indexed patterns that are most obvious, and the small fraction that go through as correct do not tend to have a high contribution since the overall averaging dilutes them. However, if the number of patterns is small, incorrectly indexed patterns should be excluded to avoid any significant biasing. In this work, the criterion was set to be when the resulting indexing (if given) provided meaningful reflection positions with respect to the experimental ones by visual inspection. In the case of *PETS2*, the refined frame scales obtained from each pattern were also checked, and those that were negative or exceedingly high were discarded. In this way, the rate of used patterns after parameter optimizations and safety checks was higher than 85% in all evaluated cases (Table 1 ▸).

**Figure 3:**
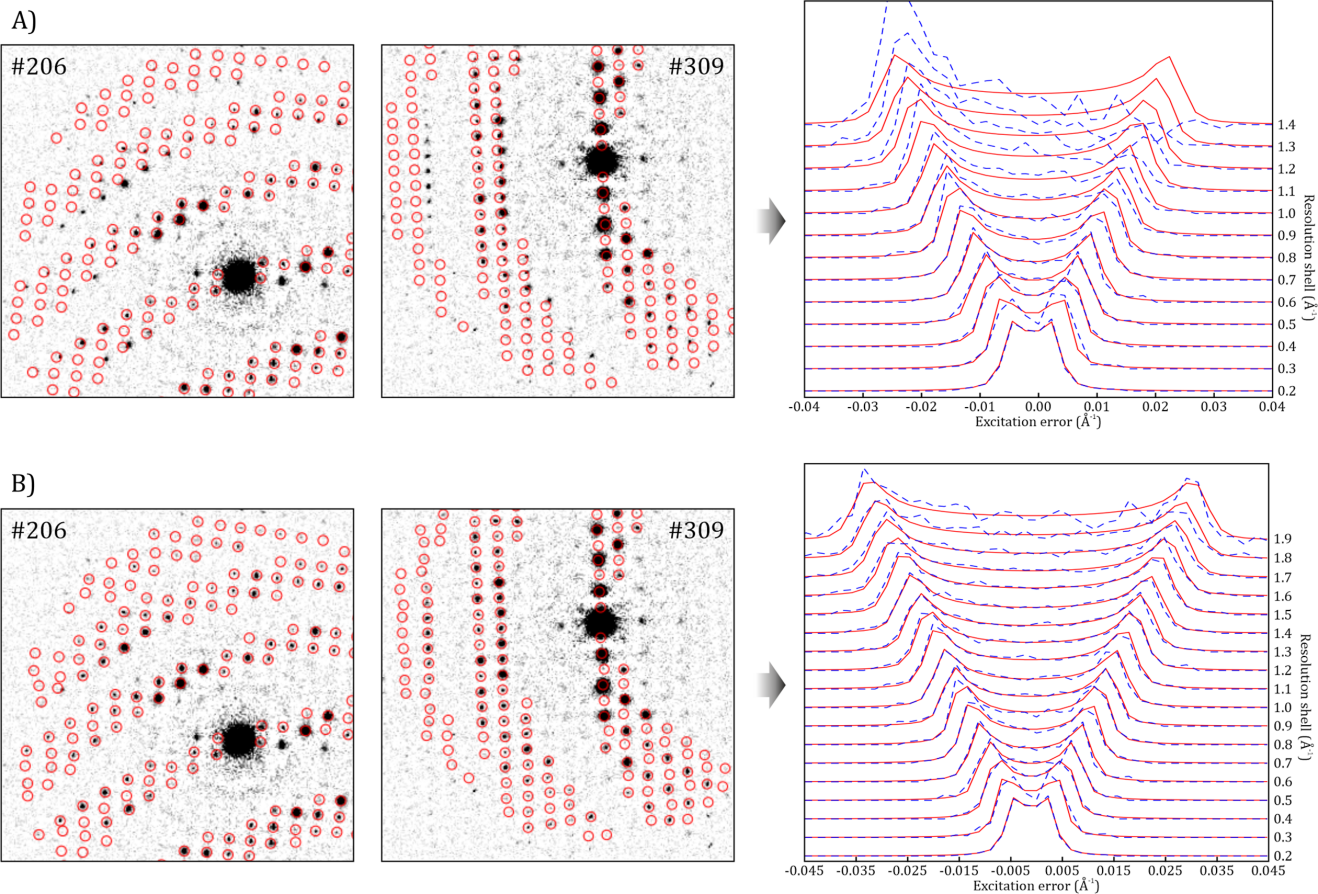
Two example background-corrected diffraction patterns of the SerialPED experiment from the Tensor microscope, with overlaid red circles that correspond to the calculated reflection positions according to the found orientation, and the resulting averaged rocking curves from *PETS2* at different resolution shells considering all diffraction patterns. Blue dashed curves represent the averaged experimental result and red dashed curves represent the simulated double-peaked curve that fits best. (*a*) Case in which reflection positions are treated as completely free of distortions and (*b*) when distortion corrections are enabled.

During the data reduction process, it was noted that the diffraction patterns acquired on the Tensor TEM exhibited a strong distortion, which was only perceptible when the calculated reflection positions according to the orientation matrix were overlapped with the experimental patterns, and the averaged rocking curves at different resolution shells were plotted for the precessed data. Fig. 3 ▸(*a*) displays two PED patterns where this mismatch can be clearly seen, together with the strong asymmetry of the double-peaked rocking curves when processing the whole respective data set. This results in very poor intensity integration for reflections far away from the central beam that leads to worse intensity statistics as the resolution increases, poor least-squares fitting of the function parameters to the experimental averaged rocking curves and thus incorrect reflection intensity extraction at the end. Unfortunately, this was not only an elliptical distortion caused by residual stigmatism of the projector system but a combination of several distortions of higher order that is suspected to be due to data acquisition in an optical plane not exactly conjugated to the back focal plane of the objective lens. In this situation, the optical distortions were corrected by applying the available option in *PETS2* (Brázda *et al.*, 2022 ▸): first, the frame-by-frame distortions that include magnification, elliptical and parabolic correcting factors were obtained by least-squares fitting on each pattern, and then the barrel-pincushion was determined by least-squares refinement on the 3D reconstruction of the 2D peak positions. The ‘radial *S*_g_ parabolic parameter’, which is related to the dependence of the parabolic distortion on the phase of the precession circuit, was also refined in the case of precessed data. These last two contributions were dominant as they reached −0.92% for static patterns, and −0.85% and −0.71%, respectively, for the precessed ones. The result of this detailed geometric correction is shown in Fig. 3 ▸(*b*), where the calculated positions of the reflections match well with the experimental ones and the double-peaked rocking curves appear symmetric up to very high resolutions. Note that such corrections are applied to the reflection positions from which the respective intensity will be integrated. Thus, no image transformation is applied to the frames, and the reconstructed observable diffraction space will still exhibit the deformation (Fig. S2). On the other hand, the *diffractem* package only has an option to correct for typical elliptical deformation; hence an equivalent data processing comparison could not be made for the serial data set from the Tensor TEM.

As mentioned above, *partialator* includes several options to merge the reflection intensities that have previously been reported not to influence the final outcome significantly (Bücker *et al.*, 2021 ▸). However, the analysis carried out here shows that the choice of these parameters for the SerialED data set determines if a successful structure solution is possible (understood as finding the maximum number of atoms), although the figures of merit (FoMs) are not good, such as negative overall atomic displacement parameters (ADPs) and high *R*_int_ values (Table S1). The case of the precessed data set is totally different as the merging is more uniform across the routines landscape (Table S2); all used merging options resulted in at least four of the five symmetry-independent atoms being found, the *R*_int_ values were significantly better than for the static case, and the overall ADPs were positive. Kinematical refinements followed for both types of serial data by using the merged .hkl file that resulted in the best performance: unity model (partialities = 1 for all reflections), Debye–Waller scaling calculation and three iterations of post-refinement.

The kinematical refinements were carried out in *JANA2020* using the structure models obtained with charge flipping, which did not appreciably change with respect to those obtained from direct methods. Tables 2 ▸ and 3 ▸ show the FoMs for the SerialED and SerialPED results, respectively. The structures could only be refined with isotropic ADPs and they became positive in all the precessed cases considered here, while some of them turned negative or were even non-refinable for the static patterns. In this context, extinction corrections were applied for the SerialED refinements to obtain positive ADPs for all atoms. Interestingly, all analyses showed that the atom that was most problematic to refine was O3, which always tended to be too close to the sulfur (below 1.3 Å), and the isotropic ADP was higher. Furthermore, the FoMs are quite high by electron diffraction standards.

**Figure 4:**
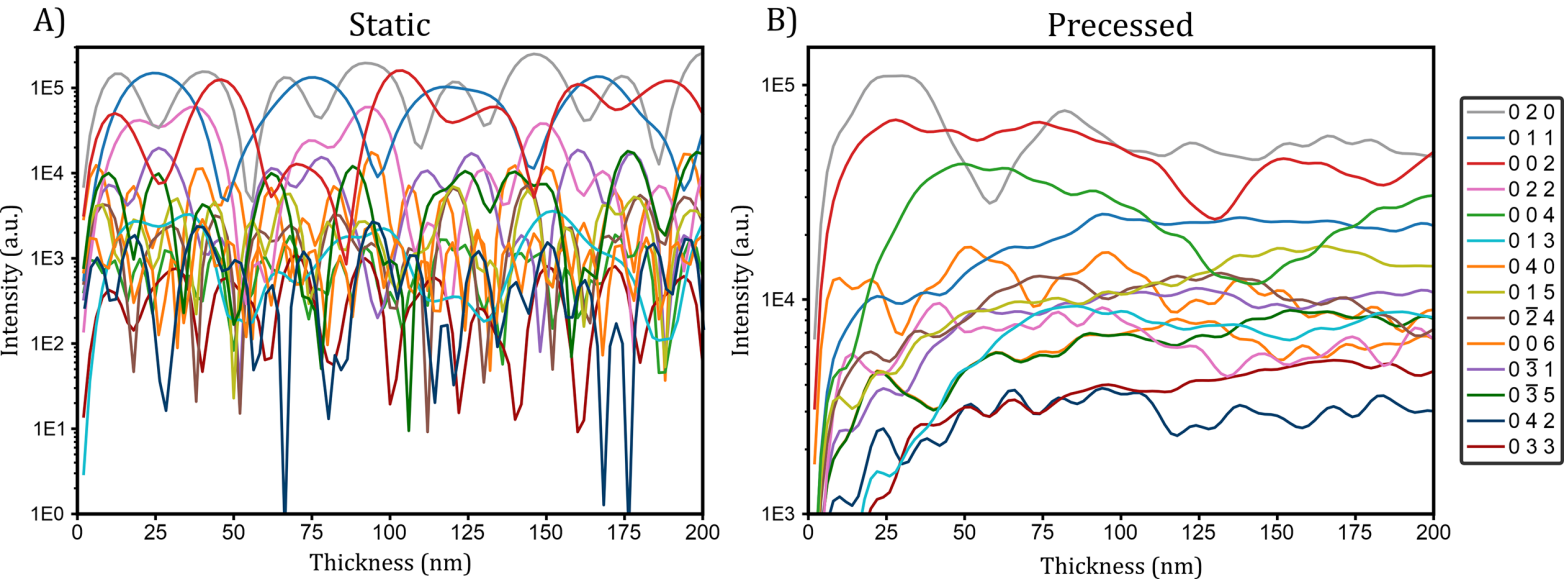
Calculated intensity values for some of the reflections present in the [100] zone-axis orientation of baryte with respect to the thickness. (*a*) The case of a static beam and (*b*) a 1° precessed beam. The Bloch wave formalism implemented in *dyngo* was used. The *y* axis is represented on a logarithmic scale and reflection intensities were calculated between thicknesses of 2 nm and 200 nm in 2 nm steps. A reflection width of 0.01 Å^−1^ and reflections up to 2 Å^−1^ were considered, leading to 96 reflections used for the calculation.

Since kinematical refinements led to a distorted tetrahedron with S—O3 distances that are too small, dynamical refinements from the precessed diffraction patterns were performed to improve the crystal structure model and the overall FoMs. Usually, such refinements can only be performed on 3D ED data sets, as the reflection intensities in the dynamical theory of diffraction are dependent on the thickness of the crystal and a single thickness value is refined for the tilt series of the individual crystal. Each pattern and its related integrated reflection intensities are treated individually, considering its crystallographic orientation and the increase in the effective thickness due to the α tilt of the sample holder (as the tilt increases, the distance that the electrons travel through the crystal increases as well). Dynamical refinements are mostly performed on ED patterns in which the collected reflection intensities have been integrated through beam precession or by rotating the goniometric stage during detector exposure. This allows a smoothing of the reflection intensity dependence on the thickness, and convergence of the structure refinement can be reached (Palatinus *et al.*, 2013 ▸). Fig. 4 ▸ shows the calculated intensities from reflections present in the [100] zone-axis orientation of baryte. The static case clearly shows the highly oscillating nature of reflection intensities, with intensity variations of one or even several orders of magnitude. The precessed reflection intensities are more uniform and the relative intensities are similar across the different thicknesses. This strong dependence that can be smeared with precession is also visible when the crystal is tilted away from highly symmetric directions (Fig. S3).

In this context, one might think of refining a thickness parameter for each pattern of the SerialPED data set. Although this should be the correct approach from a physical point of view, the refinement became unstable, as several local minima can still be found for precessed intensities and the number of reflections per pattern seems not to be high enough to reach a global minimum in the parameters landscape for convergence. To overcome this problem, the frame scales found for each diffraction pattern in *PETS2* were applied to the intensities obtained from each respective pattern. These scaling factors are calculated to minimize the intensity difference between symmetry-related reflections present in several patterns of the data set and they are usually used to generate a more suitable .hkl file for kinematical refinements. By using them for the extraction of the reflection intensities for each individual pattern in the context of a dynamical refinement, the reflection intensities across the different patterns are comparable and a single virtual thickness value can be refined for all the SerialPED data sets through the dynamical calculation procedure. Although it is not formally correct, this is presented as a successful heuristic approximation for better crystal structure refinements of serial electron diffraction data. The FoMs for the dynamical refinements following this approach from both sets of SerialPED data are shown in Table 3 ▸. From both diffraction data sets, the FoMs became significantly better in comparison to the kinematically refined structure, the geometry for the sulfur–oxygen chemical environment resembles an ideal tetrahedron, and the root-mean-square deviation (RMSD) was reduced to the picometre scales. Also, the refined virtual thickness for each SerialPED data set becomes similar, ∼55 and ∼69 nm for the Tensor and F200 microscopes, respectively.

**Figure 5:**
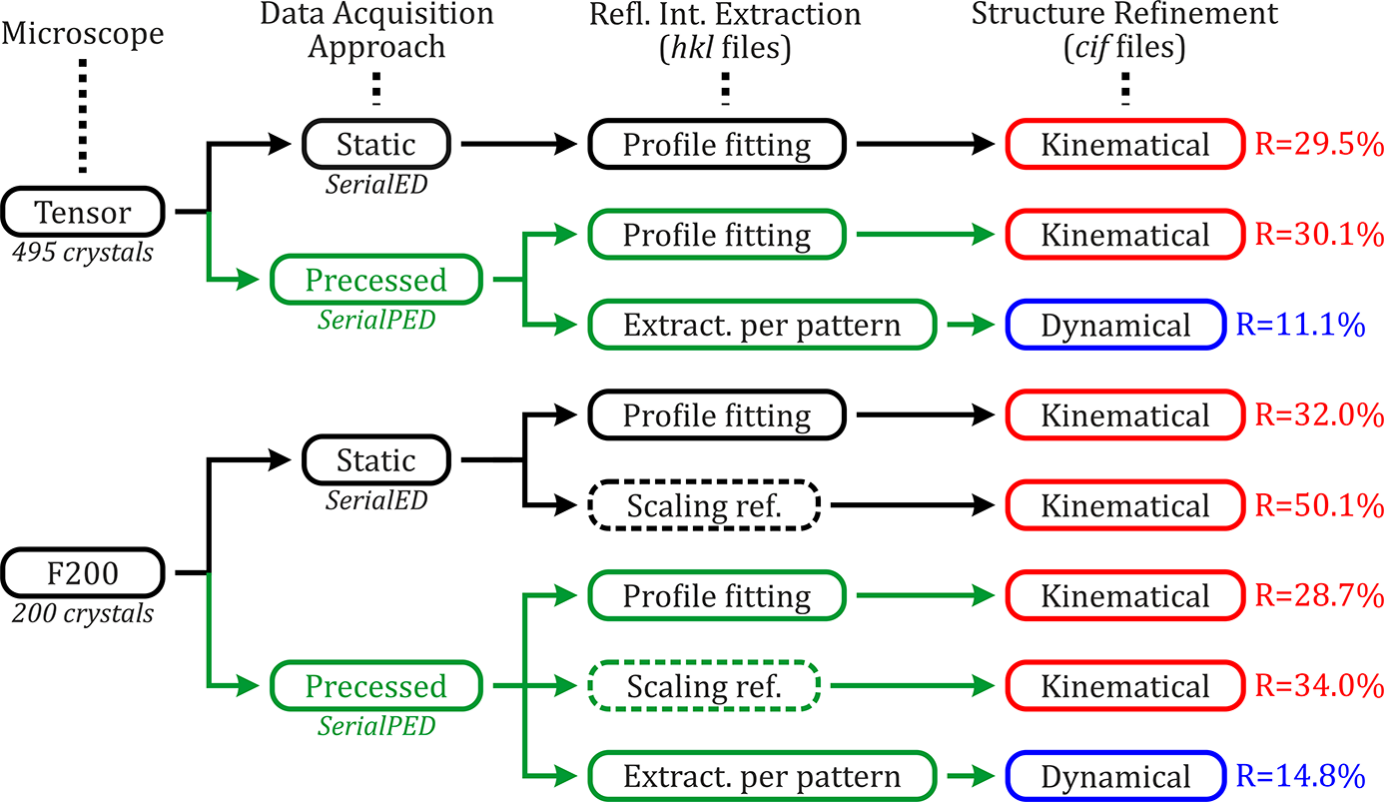
Scheme summarizing the different data processing procedures for the extraction of the reflection intensities (.hkl files) with respect to the collected serial data and the type of structure refinement carried out in each case (CIFs as final output). Kinematical refinements are highlighted in red and dynamical in blue. ‘Profile fitting’ and ‘Extract. per pattern’ reflection intensity methods were performed in *PETS2*, and ‘Scaling ref.’ (surrounded by dashed lines) in *diffractem/CrystFEL*. The *R* parameter for each structure refinement is calculated from the observed reflections up to 2 Å^−1^ / 0.5 Å resolution in *JANA2020*.

As a summary, Fig. 5 ▸ shows the different methods used for the processing of the SerialED and SerialPED data according to the microscope used and the respective type of crystal structure refinement performed for each extracted .hkl file.

## 4. Discussion

**Figure 6:**
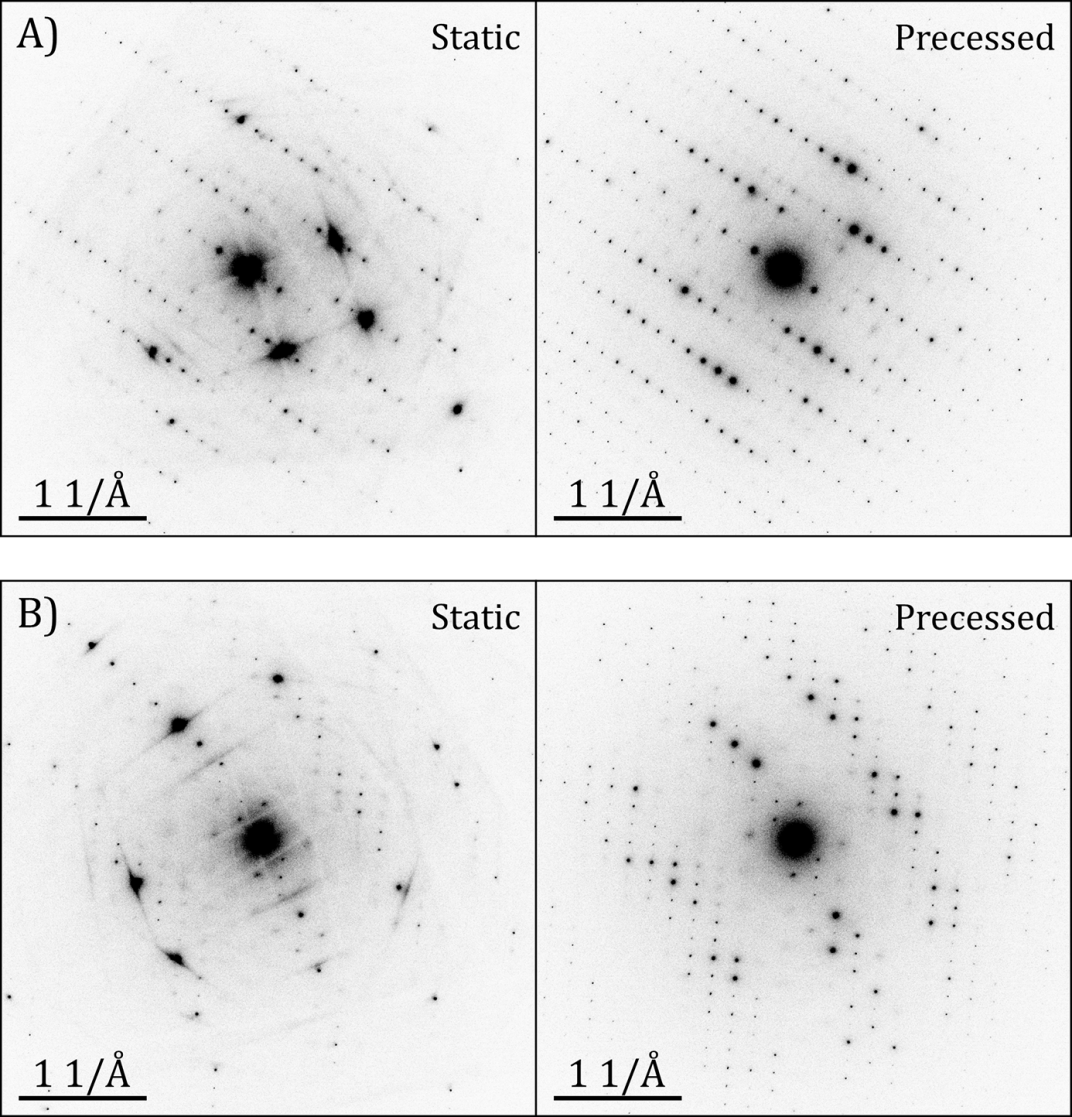
Two pairs of ED patterns from baryte crystals without (static) and with 0.92° of precession (precessed), where the effect of precession on the quality of the reflection intensities is directly visible. The displayed contrast on all patterns is the same to give the most suitable comparison.

The case study presented here aims to assess whether precession helps to improve the quality of SerialED data, and thus the crystal structure determination and refinement thereafter. Previous work focused on highly symmetric structures where the aim was to collect enough ED data before the crystalline integrity of the material vanished (Bücker *et al.*, 2020 ▸) and to demonstrate the higher resolution that one can achieve in comparison to tilt-series experiments (Hogan-Lamarre *et al.*, 2024 ▸). In this context, the analysis of a lower-symmetry inorganic crystal like BaSO_4_ becomes relevant because it allows the exploration of the scenario of serial crystallography with a low number of patterns, and hence fewer symmetrically related reflections that will be merged together. The results show that this is critical in the case of SerialED data, where both *R*_int_ and *R*_rim_ become significantly better for the set of 495 patterns (∼23% and ∼38%, respectively) than for the 200 frame data set (∼38% and ∼94%, respectively), independent of the merging protocol used. The residual electrostatic potentials from the difference Fourier maps of the kinematically refined structures are reduced by around a factor of 2 (Fig. S4). Therefore, the idea that merging diffraction data from different crystals reduces the dynamical effects, which include not only reflection intensity redistribution by multiple scattering but also Kikuchi line contributions, and enables a list of pseudo-kinematical reflections becomes directly apparent. On the other hand, the use of precession achieves this same situation in each individual pattern, reducing the required number of crystals to be measured for reliable structure analysis. Fig. 6 ▸ shows the strong influence of dynamical effects on the reflections and the significant minimization of these effects when precession is applied to the same crystal.

One of the advantages of integrating a volume from the observable diffraction space into an ED pattern by beam precession is the possibility of performing much more accurate refinements using the dynamical theory of diffraction. Minimizing the dynamical effects does not mean that reflection intensities are not intrinsically dynamical anymore, and the procedure followed here for the dynamical refinements demonstrates how the models become more accurate and reliable. The geometric similarity between the reference X-ray model and the found ED structures is much better from the dynamical refinement than for the kinematical case (RMSD reduced by an order of magnitude), and the ADPs could be anisotropically refined, resulting in positive values for all diagonal elements. The kinematical refinements could only be performed with isotropic ADPs. Moreover, the level of noise as residual electrostatic potential in the difference Fourier maps is reduced compared with the kinematic case (Fig. S4). Finally, the overall FoMs become significantly better, with the *R* and *R*_w_ figures decreased by more than a factor of 2, from ∼31% to ∼13% for *R*, and from ∼39% to ∼14% for *R*_w_ (on average for observed reflections).

**Figure 7:**
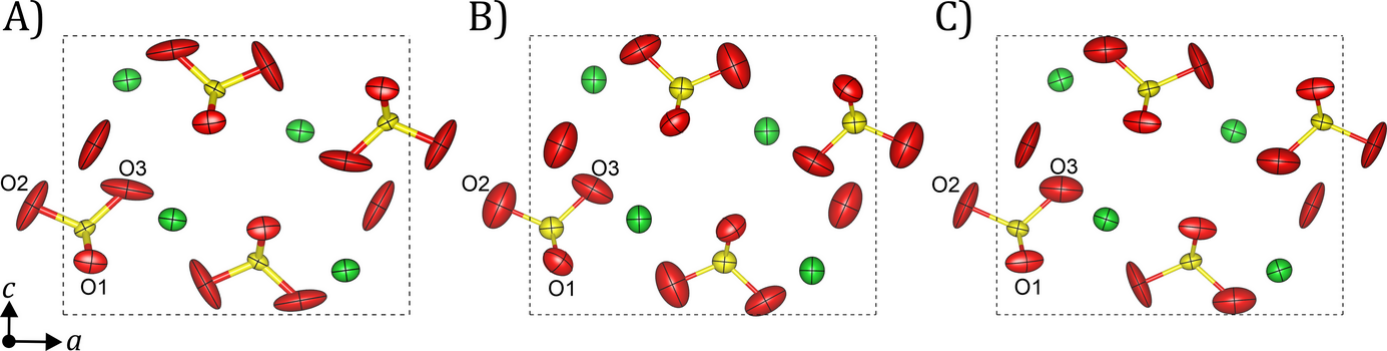
Dynamically refined structure models of baryte projected along **b** from precessed (*a*) serial and (*b*)–(*c*) tilt-series 3D ED data. Diffraction data collected on the F200 microscope on the same TEM grid, under the same illumination conditions and with the same detector parameters. The model in panel (*b*) was obtained from 121 diffraction patterns expanding 120° of angular range, while the model in panel (*c*) corresponds to 101 patterns across 100°.

Preferred orientation was noted on the reconstructed 0*kl* and *h*0*l* sections of the observable diffraction space (Fig. S2), but the completeness was high enough to avoid significant missing wedge effects on the retrieved electrostatic potential. Nevertheless, elongation of the anisotropic ADPs was observed along the **c** direction corresponding to the main direction of the missing wedge. To discount the possibility that such an effect is the result of any inappropriate data processing step, tilt-series diffraction data were collected on two different crystals from the same grid as used for the serial acquisition, and crystal structure determinations and dynamical refinements followed using the usual procedure (Table S3). Interestingly, the refined thickness parameters obtained from these refinements (∼50 and ∼57 nm) are comparable to the resulting virtual thicknesses of the SerialPED data sets, converging almost to the same averaged value for the Tensor data set. Fig. 7 ▸ shows the refined structure models along **b** for comparison. In all cases, the trend of the ADP geometry is very similar, and the strong anisotropy obtained from the serial data is also visualized in the model of Fig. 7 ▸(*c*). The inspection of the diffraction space confirmed that the missing wedge is comparable in both cases, hence the similarity in the resulting crystal structures (Fig. S5). On the other hand, the diffraction space of the model in Fig. 7 ▸(*b*) has a reduced missing wedge due to the high angular range, which explains the closer isotropy of the refined ADPs. This comparison between 3D ED acquisition techniques demonstrates the validity of the presented dynamical refinement approach on serial data.

Although it is interesting to consider the different parameters for the merging of reflection intensities in the context of kinematical diffraction, this work has found that the fluctuating intensities given by the dynamical nature of electron diffraction has a strong effect (Table S1). Furthermore, the case of a low number of patterns and relatively low symmetry implies that most reflections may not be detected more than once, and for static ED patterns, the geometric model and post-refinement merging iterations play a role in finding the best way to merge them. If the frame scales per pattern obtained by *PETS2* are compared between static and precessed diffraction data sets, the difference between the (P)ED patterns can be roughly quantified, which demonstrates the suitability of precession in these situations. For SerialED, the mean frame scales were 0.92 ± 0.68 and 0.98 ± 0.63 for the Tensor TEM and F200 TEM, respectively. In SerialPED, they become 1.00 ± 0.54 for the former and 1.00 ± 0.28 for the latter. The standard deviation is smaller in both cases, indicating the presence of more uniform reflection intensities across the ED patterns acquired with precession. This can also be visualized on the averaged rocking curves, which become noisier, and the profile fitting is worse for the static data collection (Fig. S6).

The use of different intensity extraction and indexing algorithms allowed the evaluation of different data processing pipelines. The main significant difference is found for static data. Here, the profile fit resulted in a lower number of successfully indexed patterns and a lower number of merged reflections (Table 1 ▸). Completeness is thus lower as well, but the final *R*_int_ and *R*_rim_ are better than for the scaling refinement of *partialator* (Table 1 ▸). The FoMs are also better for the profile fit, but the respective refined structure converged with Ba, S and one O with negative ADPs if an extinction correction was not applied to the observed reflection intensities. Note that the modified Monte Carlo approach of *CrystFEL* relies on a large number of partially integrated reflections rather than a few in order to extract their intensities properly, and hence it is not optimized for this type of diffraction data. The kinematical refinement also shows that the number of reflections above 3σ is very low for the profile fit, which could be explained by the high standard uncertainties of the poor fitting of the function obtained from the averaged rocking curves to the experimental rocking curves of each reflection (Fig. S6) (Palatinus *et al.*, 2019 ▸). Ideally, the frame scales should compensate for the large variation in reflection intensities between the patterns. Since the reflection intensities of each pattern are multiplied by the respective frame factor to then calculate the averaged rocking curves, the fluctuations in the curves should be smoothed out. However, the high dynamical effects present in the ED data prevent this and result in high σ values for the reflections. From the precession point of view, *PETS2* considers the geometry of precession to calculate the position of reflections that should be visible in each diffraction pattern, which results in more extracted reflection intensities per pattern than for the static case. *indexamajig* does not take it into account and the only way to induce the software to consider more reflections in each pattern for their intensity extraction is to increase the reflection profile radius, yet this is still far away from the number of reflections considered by a precession experiment. However, the kinematically refined structures are very similar in terms of FoMs and ADPs (Table 3 ▸). The indexing algorithms did not perform differently either; the only key aspect is the correct pixel calibration (Å^−1^ per pixel) with respect to the given unit-cell parameters, which strongly determines successful indexing, and both pipelines incorporate tools to optimize and/or refine parameters to increase the respective score functions.

Finally, one of the strong aspects of *PETS2* is the correction of optical distortions on the ED data (Brázda *et al.*, 2022 ▸). This is an important step, as it is very common to acquire ED patterns in an optical plane that is not exactly conjugated to the back focal plane of the objective lens, for instance by not using the standard lens currents of the objective and diffraction lenses. It becomes crucial for correct reflection integration as the resolution increases, which is certainly key for the structure determination of complex compounds. One may wonder whether the observed distortions could affect the focusing of the reflections when precession is used, *i.e.* whether reflection splitting could be problematic. If one does not consider applying nonlinear offsets at the different phases of the sinusoidal signals of precession (Viladot *et al.*, 2013 ▸), it comes down to the number of pixels and the point-spread function of the detector, as well as the effective camera length. This means that, given the same diffractive object and the same effective camera length (equal resolution at the edge of the detector), the intensity counts for a given reflection will be spread around more pixels on detectors with a higher number of pixels. Thus, the possibility of observing splitting is higher for these detectors. In this work, the pixel calibration for the optical fibre-coupled detector was 0.001 Å^−1^ and for the direct detector it was 0.011 Å^−1^, extending the intensity for a given reflection on a circular area of around 50 pixels in diameter for the former and 5–6 pixels for the latter. Therefore, if splitting occurs with a maximum deviation of 0.01 Å^−1^, it will be seen with the indirect detector as it will represent an elongation of the reflection intensity of ∼10 pixels in a specific direction, while it will not be observed with the direct detector as all electrons with such angular spread will fall on the same pixel (for simplicity, the point-spread function has not been considered). Nonetheless, a careful optical alignment of the electron beam should always be carried out to avoid such distortions as much as possible and gain the most from the diffraction space.

## 5. Conclusions

The thorough investigation carried out here using ED data sets of BaSO_4_ shows that the use of precession in a serial electron crystallography experiment is advantageous. The presented work demonstrates that PED helps to extract pseudo-kinematical reflection files with fewer diffraction patterns. The requirement for a high number of patterns in a typical SerialED acquisition is mandated by proper sampling of partially integrated reflections and a reduction in the dynamical effects through averaging across the serial data set, but the use of precession accomplishes this minimization in each pattern, hence reducing the number of crystals to be measured. In the past, such a line of thought was applied to zone-axis patterns, where precession helped to diminish the dynamical effects, but it was not always enough to have a successful structure determination from the merging of a few patterns following the kinematical approach (Gjønnes *et al.*, 1998 ▸). Combining a few hundred randomly oriented crystals with precession seems to be the ideal experimental setup for crystal structure analysis. Furthermore, dynamical refinements become possible due to the integration of a small fraction of the diffraction space into each individual pattern, which can be performed to increase the accuracy of the structure models by an order of magnitude, similar to what was observed with tilt-series 3D ED data (Palatinus, Corrêa *et al.*, 2015 ▸; Klar *et al.*, 2023 ▸).

The lowering of the required number of diffraction patterns for successful and accurate structure determinations opens the possibility to use such a data acquisition methodology in other laboratories that do not have automated collection protocols. This type of data can be acquired in a reasonable amount of time following a semi-automated method. The stage does not have to be optimized for tomography (tilt or rotation) experiments, expanding the range of microscopes that can be utilized for this purpose.

These findings will be of special benefit to the investigation of beam-sensitive materials such as metal–organic frameworks, molecular crystals, or even macromolecules that cannot be synthesized in very large amounts and which withstand only very low electron dose levels. Following the STEM acquisition protocol given in Fig. 1 ▸, the illumination of promising crystals is completely minimized until the actual acquisition of the ED pattern. Typical pixel dwell times with STEM detectors are in the microsecond regime, which leads to an electron fluence per reference scanned image of less than 0.1 e^−^ Å^−2^ (Plana-Ruiz *et al.*, 2018 ▸; Gallagher-Jones *et al.*, 2019 ▸). This provides the opportunity to expose targeted nanocrystals to an intense beam in order to collect ED patterns with high signal-to-noise reflection intensities, even though crystallinity will be lost after exposure in a similar fashion to serial crystallography in XFELs. In this way, SerialPED is presented as a reliable alternative approach for structural analysis based on electron diffraction.

## Supplementary Material

Additional tables and figures. DOI: 10.1107/S1600576725005606/yr5158sup1.pdf

CIFs and refinement files. DOI: 10.1107/S1600576725005606/yr5158sup2.zip

## Figures and Tables

**Table 1:** Data reduction statistics for the Serial(P)ED data collected from baryte crystals on the different microscope setups Data processing according to ‘Profile fit’ was obtained from *PETS2*, while ‘Scaling refinement’ was done through *diffractem*/*CrystFEL* with three post-refinement iterations. ‘Used patterns’ refers to the number of patterns that have been correctly indexed and ‘Refls’ stands for reflections. ‘Integrated Refls’ represents the total number of integrated reflections through the whole serial data set without merging. Reflections up to 2 Å^−1^ / 0.5 Å resolution have been considered.

Microscope	F200				Tensor	
Reflection intensity extraction	Profile fit		Scaling refinement		Profile fit	
Data acquisition approach	Static	Precessed	Static	Precessed	Static	Precessed
Used patterns	174	199	193	189	472	485
Percentage of all patterns (%)	87.0	99.5	96.5	94.5	95.4	98.0
Integrated Refls	20035	53264	20706	22719	28310	129939
Merged Refls	3067	8258	8838	9437	3970	9227
Independent Refls†	1083	1478	1320	1364	960	1477
Completeness (%)†	70.01	95.54	85.33	88.17	62.14	99.06
*R*_int_ (%)†	31.96	11.60	44.44	16.76	23.31	13.07
*R*_rim_ (%)‡	58.83	20.41	129.39	36.66	39.33	17.91

† As calculated by *SIR2014* for reflections with intensity above 3σ(*I*).

‡ As calculated by *XPREP* for all reflections.

**Table 2:** Figures of merit for the structure refinements carried out in *JANA2020* for the SerialED data collected from baryte crystals on the different microscope setups ‘Profile fit Int.’ stands for profile fit intensity extraction of the .hkl file obtained from *PETS2*, while ‘Scaling ref. Int’ stands for scaling refinement intensity extraction of the .hkl file from *diffractem*/*CrystFEL* using three post-refinement iterations. RMSD corresponds to the root-mean-square deviation between the atom positions of the asymmetric unit of the refined model and the ones from the structure model of Jacobsen *et al.* (1998 ▸) as reference, and ‘Max. deviation’ refers to the maximum distance variation of these (both parameters calculated by *SIR2014*). An isotropic extinction correction in the form of a Becker–Coppens type 1 Gaussian was used during the refinements (Becker & Coppens, 1974 ▸). The number of reflections, goodness of fit, and *R* and *R*_w_ parameters are calculated and reported from observed and all (obs/all) reflections up to 2 Å^−1^ / 0.5 Å resolution. The criterion for observed reflections was *I*(*h*) > 3σ(*h*).

Microscope	F200 (200 kV)		Tensor (100 kV)
Refl. intensity extraction	Scaling ref. Int.	Profile fit Int.	Profile fit Int.
Number of reflections	2281/2717	267/1083	431/1180
Reflections/parameters	126.7	14.8	23.9
Goodness of fit (%)	41.40/37.93	3.16/2.19	3.62/2.63
*R* (%)	50.10/52.45	32.04/41.12	29.50/36.85
*R*_w_ (%)	55.05/55.08	36.19/42.74	35.50/39.38
RMSD† (Å)	0.098	0.053	0.064
Max. deviation (Å)	0.425 (16)	0.24 (3)	0.27 (5)

† Calculated as √(1/*N* ∑_*i*=1_^*N*^ |*r*_*i*_ − *r*_*i*_^ref^|^2^).

**Table 3:** Figures of merit for the structure refinements carried out in *JANA2020* for the SerialPED data collected from baryte crystals on the different microscope setups ‘Profile fit Int.’ stands for profile fit intensity extraction of the .hkl file obtained from *PETS2*, while ‘Scaling ref. Int’ stands for scaling refinement intensity extraction of the .hkl file from *diffractem*/*CrystFEL* using three post-refinement iterations. RMSD corresponds to the root-mean-square deviation between the atom positions of the asymmetric unit of the refined model and those from the structure model of Jacobsen *et al.* (1998 ▸) as reference, and ‘Max. deviation’ refers to the maximum distance variation of these (both parameters calculated by *SIR2014*). The number of reflections, goodness of fit, and *R* and *R*_w_ parameters are calculated and reported from observed and all (obs/all) reflections up to 2 Å^−1^ / 0.5 Å resolution. The criterion for observed reflections was *I*(*h*) > 3σ(*h*). The ‘Reflections/parameters’ ratio refers to the number of observed reflections over the number of refined parameters. *R* and *R*_w_ are based on the square root of the reflection intensities. Dynamical refinements were executed with *g*_max_ of 2.2 Å^−1^, *S*_*g*_^max^(matrix) of 0.01 Å^−1^, *S*_*g*_^max^(refine) of 0.1 Å^−1^, *RS_g_* of 0.66, and *N*_or_ of 83 for the F200 data and 87 for the Tensor data.

Microscope	F200 (200 kV)			Tensor (100 kV)	
Refinement type	Kinematical (Scaling ref. Int.)	Kinematical (Profile fit Int.)	Dynamical	Kinematical	Dynamical
Number of reflections	2127/2818	1194/1471	19257/20891	1190/1477	16602/53534
Reflections/parameters	125.1	70.2	83	70.0	32.1
Goodness of fit (%)	28.94/25.15	6.19/5.65	7.77/7.47	8.04/7.27	2.52/1.56
*R* (%)	34.04/36.30	28.66/30.12	14.77/15.23	30.05/32.13	11.07/20.71
*R*_w_ (%)	43.36/43.38	36.22/36.50	17.01/17.04	38.29/38.46	11.70/12.81
RMSD (Å)†	0.072	0.047	0.008	0.076	0.010
Max. deviation (Å)	0.34 (3)	0.21 (3)	0.022 (4)	0.23 (10)	0.035 (3)

† Calculated as √(1/*N* ∑_*i*=1_^*N*^ |*r*_*i*_ − *r*_*i*_^ref^|^2^).

## Data Availability

All raw diffraction patterns from the serial and tilt-series 3D ED experiments used in this work can be found on Zenodo through the following link: https://zenodo.org/records/14143495.
