# Supplementary material for: On the use of beam precession for serial electron crystallography
Source: J Appl Crystallogr. 2025 Jul 25;58(Pt 4):1249–60. doi: 10.1107/S1600576725005606 (PMC12321033; doi:10.1107/S1600576725005606)
Supplement: Supplementary file 1 [file j-58-01249-sup1.pdf]

JOURNAL OF
APPLIED
CRYSTALLOGRAPHY

Volume 58 (2025)

Supporting information for article:

On the use of beam precession for serial electron crystallography

Sergi Plana-Ruiz, Penghan Lu, Govind Ummethala and Rafal E. Dunin-Borkowski

Table S1 Merging statistics and results from *ab initio* structure solutions (*Sir2014*) of the SerialED data collected from baryte crystals on the F200 TEM according to the different options of *partialator* in the *CrystFEL* software package. "PR ite." stands for post-refinement iterations, "Independent Refls" for symmetrically independent reflections, and "Compl." for completeness. Overall ADP is the isotropic atomic displacement parameter obtained from the Wilson plot and given as B. Reflections up to 2 Å^{-1} / 0.5 Å resolution have been considered.

PR ite.	Partiality model	Debye Waller calculation	Independent Refls*	R_{int}*(%)	Compl.* (%)	Overall ADP* (Å^2)	Found atoms from direct methods
0	*Offset*	No	1220	35.11	78.86	-0.090	Ba, S, O1, O2
		Yes	1230	36.04	79.51	-0.069	Ba, S, O1, O2
	Unity	No	1325	44.00	85.65	-0.104	Ba, S, O1, O2, O3
		Yes	1342	44.55	86.75	-0.071	Ba, S, O1, O2
	Xsphere	No	1193	35.60	77.17	-0.225	Ba, S
		Yes	1194	37.01	77.23	-0.392	Ba, S, O1, O2
1	*Offset*	No	1221	34.03	78.93	-0.106	Ba, S, O1, O2
		Yes	1233	34.94	79.9	-0.67	Ba, S, O1
	Unity	No	1323	44.16	85.52	-0.113	Ba, S, O1, O2
		Yes	1325	44.83	85.65	-0.010	Ba, S, O1
	Xsphere	No	1171	34.15	75.69	-0.129	Ba, S, O1
		Yes	1192	36.9	77.05	-0.416	Ba, S, O1
3	*Offset*	No	1206	33.81	77.96	-0.151	Ba, S, O1
		Yes	1206	34.64	77.96	-0.162	Ba, S, O1
	Unity	No	1291	44.30	83.45	-0.099	Ba, S, O1, O2, O3
		Yes	1320	44.44	85.33	-0.018	Ba, S, O1, O2
	Xsphere	No	1109	33.06	71.69	-0.15	Ba, S
		Yes	1163	36.85	75.18	-0.467	Ba, S, O1

* As calculated by *Sir2014* for reflections that fulfil that their intensity is above 3σ(I).

Table S2 Merging statistics and results from *ab initio* structure solutions (*Sir2014*) of the SerialPED data collected from baryte crystals on the F200 TEM according to the different options of *partialator* in the *CrystFEL* software package. "PR ite." stands for post-refinement iterations, "Indepdendent Refls" for symmetrically independent reflections, and "Compl." for completeness. Overall ADP is the isotropic atomic displacement parameter obtained from the Wilson plot and given as B. Reflections up to 2 Å^{-1} / 0.5 Å resolution have been considered.

PR ite.	Partiality model	Debye Waller calculation	Independent Refls*	R_{int}*(%)	Compl.* (%)	Overall ADP* (Å^2)	Found atoms from direct methods
0	*Offset*	No	1272	18.90	82.22	0.630	Ba, S, O1, O2
		Yes	1249	18.79	80.74	0.598	Ba, S, O1, O2
	Unity	No	1364	16.48	88.17	0.673	Ba, S, O1, O2
		Yes	1364	16.77	88.17	0.669	Ba, S, O1, O2
	Xsphere	No	1214	17.84	78.47	0.624	Ba, S, O1, O2
		Yes	1191	18.95	76.99	0.600	Ba, S, O1, O2
1	*Offset*	No	1256	18.63	81.19	0.654	Ba, S, O1, O2
		Yes	1229	18.82	79.44	0.684	Ba, S, O1, O2
	Unity	No	1364	16.5	88.17	0.673	Ba, S, O1, O2
		Yes	1364	16.78	88.17	0.691	Ba, S, O1, O2
	Xsphere	No	1199	17.91	77.5	0.651	Ba, S, O1, O2
		Yes	1176	18.31	76.02	0.668	Ba, S, O1, O2
3	*Offset*	No	1209	18.76	78.15	0.674	Ba, S, O1, O2, O3
		Yes	1218	18.87	78.73	0.714	Ba, S, O1, O2
	Unity	No	1364	16.5	88.17	0.673	Ba, S, O1, O2, O3
		Yes	1364	16.76	88.17	0.744	Ba, S, O1, O2
	Xsphere	No	1187	17.95	76.73	0.606	Ba, S, O1, O2
		Yes	1136	18.68	73.43	0.703	Ba, S, O1, O2

* As calculated by *Sir2014* for reflections that fulfil that their intensity is above 3σ(I).

Table S3 Data reduction statistics and figures-of-merit for the dynamical refinements carried out in *Jana2020* for the tilt-series 3D ED data of two baryte crystals.

	**Crystal 1**	**Crystal 2**
Angular range (°)	120	100
Number of patterns	121	101
Precession angle (°)	1.086	1.086
Integrated Refls	37142	31602
Merged Refls	7531	6395
Independent Refls*	1520	1216
Completeness* (%)	98.25	78.6
R_{int}* (%)	10.94	10.49
Number of reflections	14140/16149	11880/13025
Reflections/Parameters (-)	91.2	88.0
GoF (%)	5.88/5.52	6.20/5.93
R (%)	13.48/14.02	13.94/14.28
R_w (%)	15.30/15.34	15.73/15.75
Refined thickness (Å)	502.1	573.3

* As calculated by *Sir2014* for reflections that fulfil that their intensity is above 3σ(I). The number of reflections, goodness of fit (*GoF*), R and R_w parameters are calculated and reported from observed and all (obs/all) reflections up to 2 Å^{-1} / 0.5 Å resolution. The criterion for observed reflections was I(**h**) > 3σ(**h**). The 'Reflections/Parameters' ratio refers to the number of observed reflections over the number of refined parameters. R and R_w are based on the square root of reflection intensities. Dynamical refinements were executed with g_{max} of 2.2 Å^{-1}, S_g^{max}(matrix) of 0.01 Å^{-1}, S_g^{max}(refine) of 0.1 Å^{-1}, RS_g of 0.66, and N_{or} of 94 for crystal 1 and 98 for crystal 2.

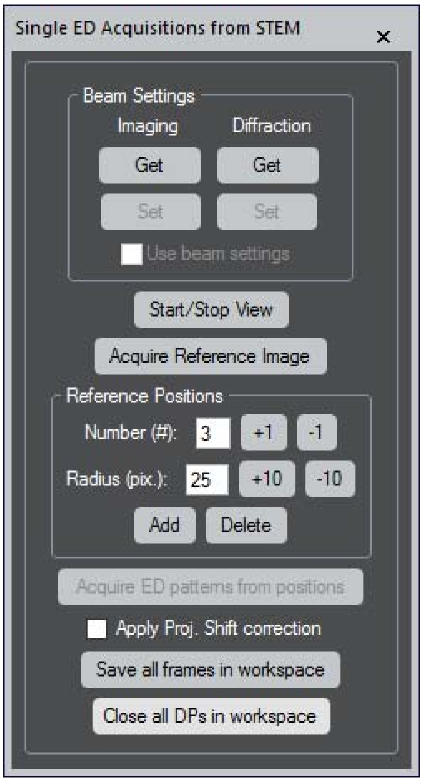

Figure S1 Screenshot of the graphical user interface for the SerialED data collection developed in the Gatan Digital Micrograph environment.

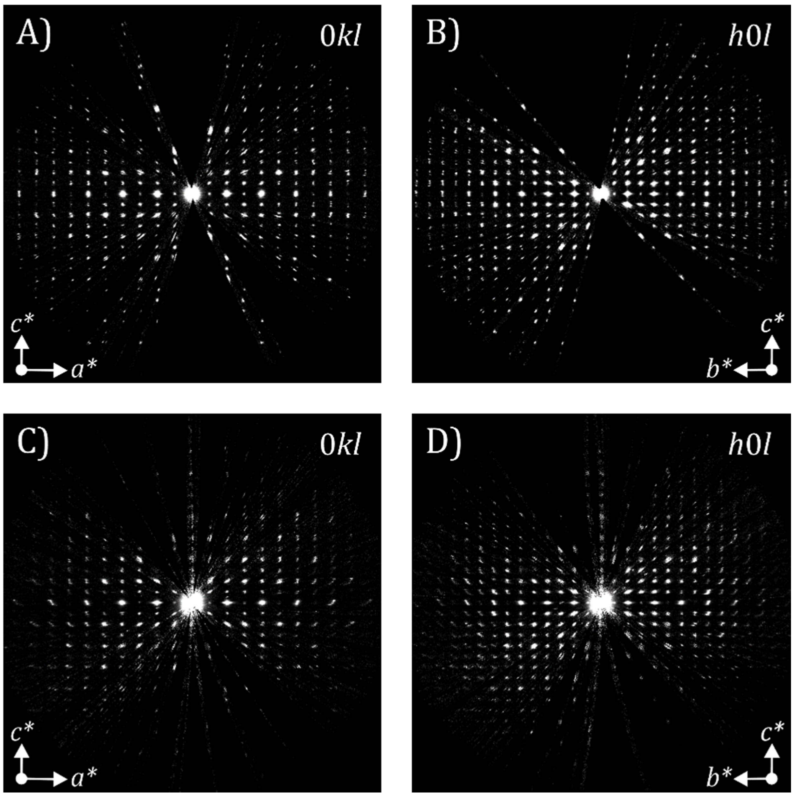

Figure S2 $0kl$ and $h0l$ sections of the reconstructed observable diffraction space of baryte from the SerialPED data collected on the A)-B) F200 TEM and C)-D) Tensor microscope. The low coverage along the $c*$ axis indicates the preferred orientation of the crystals. Sections calculated with *PETS2* according to the found indexing.

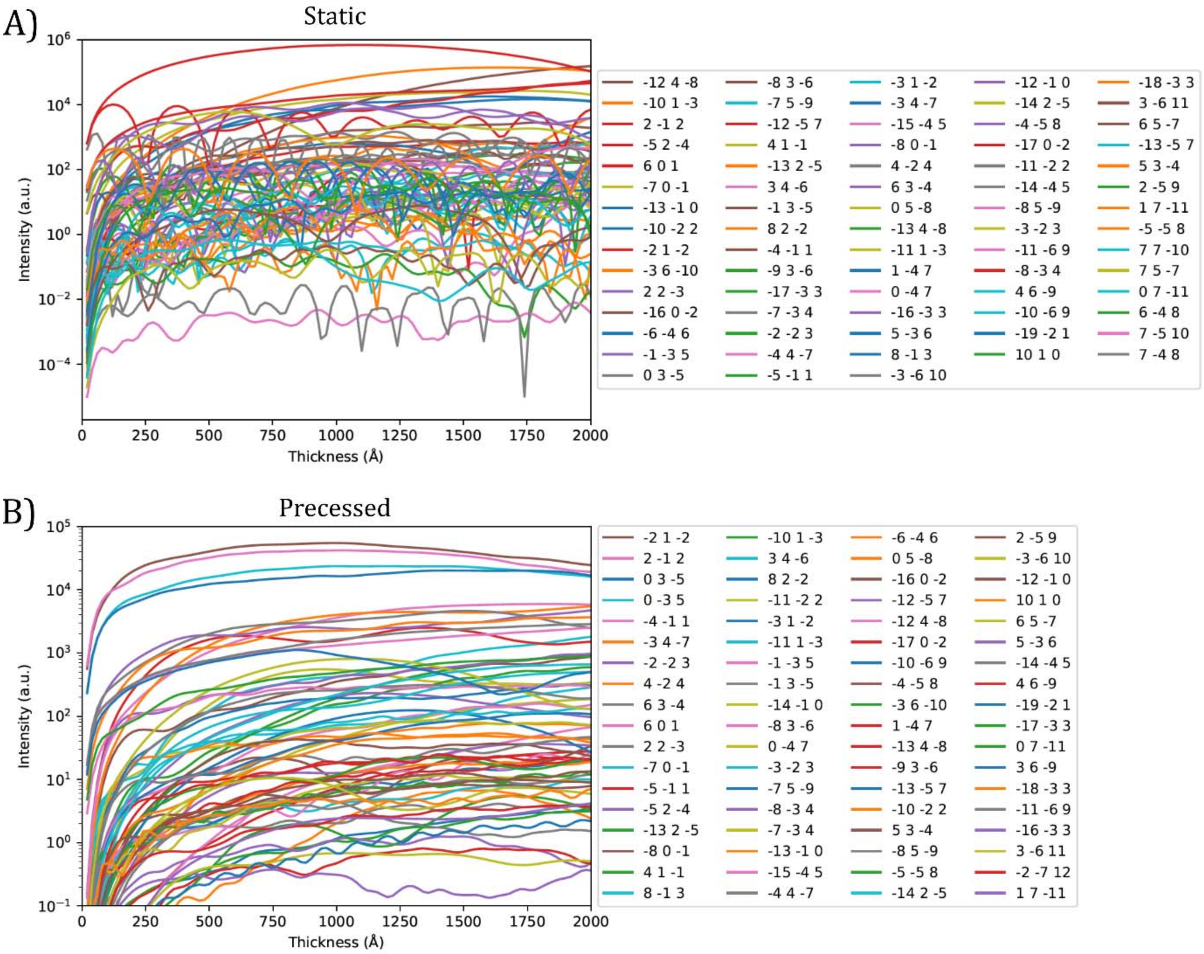

Figure S3 Intensity values for reflections close to the [0 10 6] zone-axis of baryte with respect to the thickness. A) corresponds to the case of a static beam while B) when an electron beam of 0.92° of precession is used. The Bloch wave formalism implemented in *dyngo* was used for this simulation. The y axis is represented in logarithmic scale and reflection intensities were calculated between thicknesses of 2 nm and 200 nm in 2 nm steps. A reflection width of 0.01 Å^{-1} and reflections up to 2 Å^{-1} were considered, leading to 87 reflections used for the calculation. The different reflections are listed according to their intensity at 2000 nm; from the strongest to the weakest.

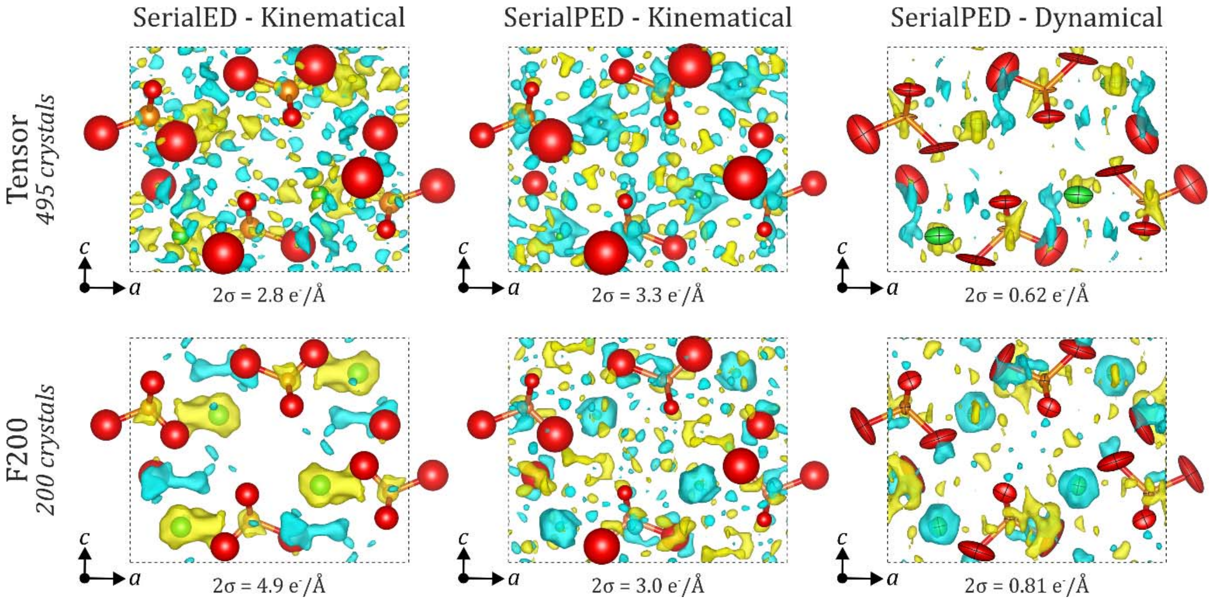

Figure S4 Structure models along the ***b*** and difference Fourier maps showing the residual electrostatic potentials at the 2σ level (in e^-/Å, Gaussian units system) for the structure refinements obtained from the reflection intensities extracted with the profile fit. Yellow potential corresponds to positive values and light blue to negative ones. Barium is represented as green, sulfur as orange and oxygen as red.

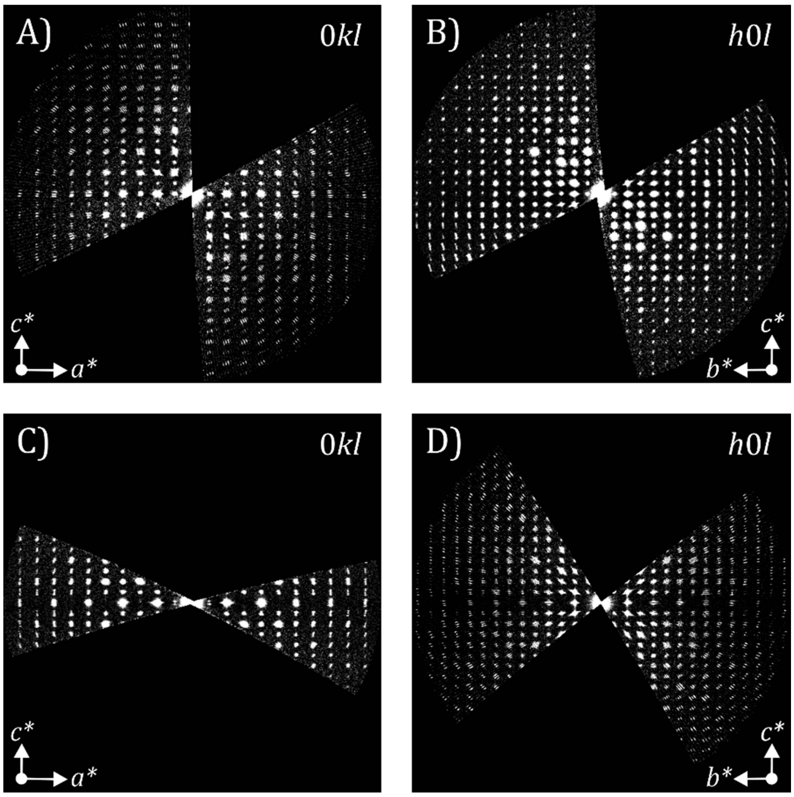

Figure S5 *hk*0and *h*0*l* sections of the reconstructed observable diffraction space of baryte from the tilt-series 3D ED data collected of A)-B) crystal 1 and C)-D) crystal 2. Sections calculated with *PETS2* according to the found indexing.

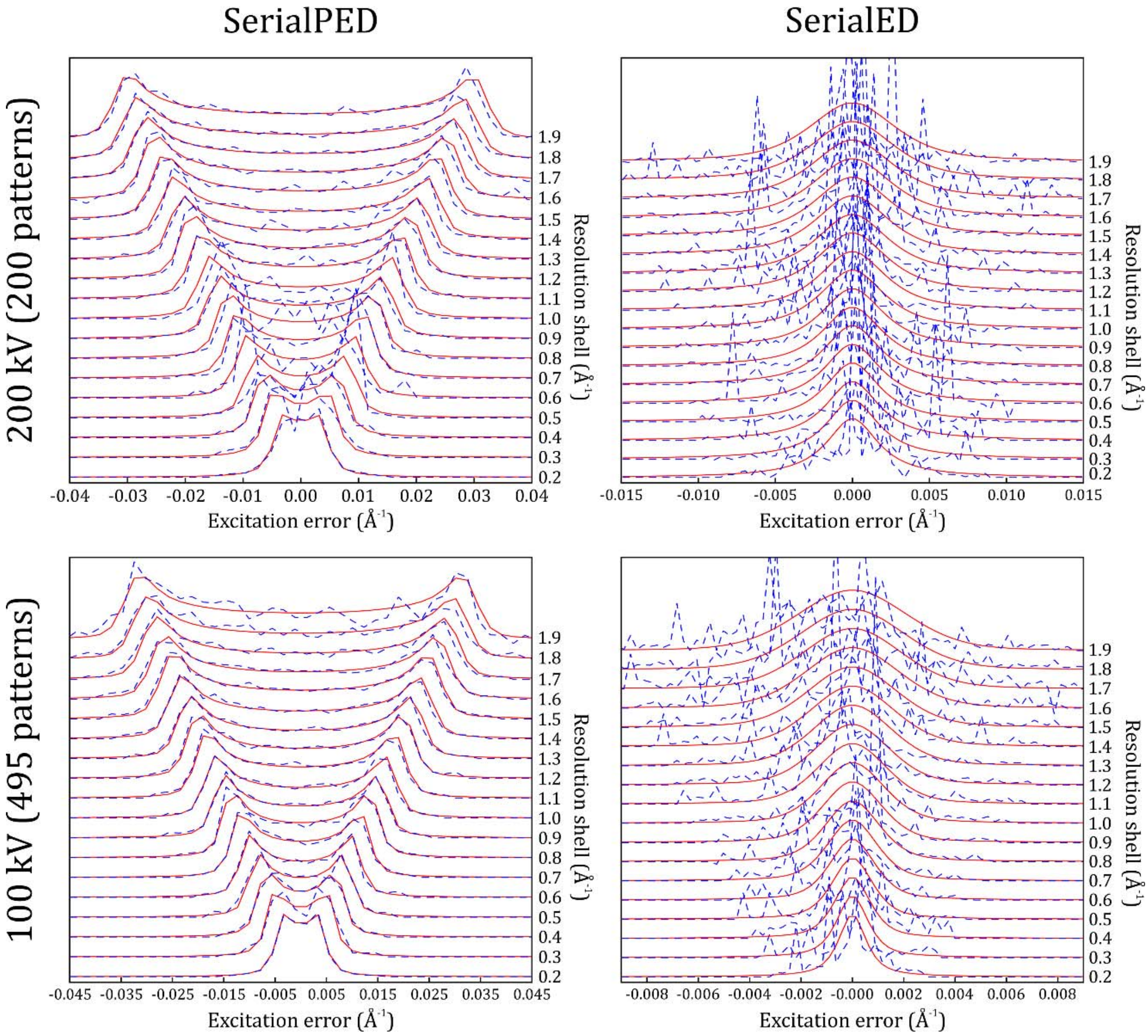

Figure S6 Averaged rocking curves at different resolution shells for the Serial(P)ED data of baryte crystals from the two different microscope setups obtained from *PETS2*. Blue-dashed curves represent the averaged experimental result and the red ones the simulated double-peaked curve that fits best.
